# Joint modelling of longitudinal and time-to-event data with application to predicting abdominal aortic aneurysm growth and rupture

**DOI:** 10.1002/bimj.201100052

**Published:** 2011-08-10

**Authors:** Michael J Sweeting, Simon G Thompson

**Affiliations:** MRC Biostatistics Unit, Institute of Public HealthRobinson Way, Cambridge, CB2 0SR, UK

**Keywords:** Abdominal aortic aneurysm, Hierarchical model, Joint model, Prediction, Shared random effects

## Abstract

Shared random effects joint models are becoming increasingly popular for investigating the relationship between longitudinal and time-to-event data. Although appealing, such complex models are computationally intensive, and quick, approximate methods may provide a reasonable alternative. In this paper, we first compare the shared random effects model with two approximate approaches: a naïve proportional hazards model with time-dependent covariate and a two-stage joint model, which uses plug-in estimates of the fitted values from a longitudinal analysis as covariates in a survival model. We show that the approximate approaches should be avoided since they can severely underestimate any association between the current underlying longitudinal value and the event hazard. We present classical and Bayesian implementations of the shared random effects model and highlight the advantages of the latter for making predictions. We then apply the models described to a study of abdominal aortic aneurysms (AAA) to investigate the association between AAA diameter and the hazard of AAA rupture. Out-of-sample predictions of future AAA growth and hazard of rupture are derived from Bayesian posterior predictive distributions, which are easily calculated within an MCMC framework. Finally, using a multivariate survival sub-model we show that underlying diameter rather than the rate of growth is the most important predictor of AAA rupture.

## 1 Introduction

In clinical trials or observational studies it is common to collect information about a time-to-event outcome, together with longitudinal measurements of a disease marker. Joint modelling has become an increasingly popular approach to characterise the relationship between the longitudinal and time-to-event processes. The literature in this area is now extensive, and good overviews can be found in Tsiatis and Davidian (2004) and Hogan and Laird (1998), with seminal papers by Tsiatis et al. (1995) and Self and Pawitan (1992) focusing on the joint modelling of CD4 counts and time to AIDS.

This paper is motivated by a project where the objective is to estimate growth and rupture rates in patients with abdominal aortic aneurysms (AAA), and to assess implications for monitoring intervals. AAAs are swellings of the aorta, which grow over time and can rupture if left untreated, leading to a high mortality rate (Bown et al., 2002). Although it has long been known that the current AAA diameter is strongly associated with the risk of rupture, the question remains as to whether the rate of AAA growth is an independent explanatory variable. In addition to this basic aetiological question, predictions of future AAA diameter and risk of rupture are required to inform appropriate monitoring intervals for screening programmes. In particular, given an individual with a series of AAA diameter measurements, an appropriate monitoring interval can be chosen based on their predicted cumulative incidence of rupture, or the decision can be taken to refer the patient for surgery if the risk of rupture before the next screen is too high. We illustrate in this paper how joint models can be used to make such predictions.

We first contrast three methods for modelling the association between aspects of a longitudinal process and a time-to-event outcome. A naïve approach, where repeated measurements are used as time-dependent covariates in a Cox (Cox, 1972) or parametric survival model, is compared to a shared random effects joint model and a two-stage joint modelling approach. Section 2 presents a classical implementation, and describes the pros and cons of each method. A simulation study is conducted in Section 3 to contrast the three approaches in their ability to estimate the true association between a longitudinal process and the event hazard. We then describe in Section 4 how a shared random effects joint model can be fit using Bayesian MCMC methods in WinBUGS (Spiegelhalter et al., 2003). Such software is flexible in allowing a number of possible associations between the longitudinal and time-to-event processes to be investigated, while predicted survival curves and future longitudinal values can easily be obtained for an individual using posterior predictive distributions. Nevertheless, the Bayesian model implemented in WinBUGS is currently restricted to models in which the cumulative hazard has a closed form. Finally, in Section 5 we apply the models to data from the Multicentre Aneurysm Screening Study, which has collected repeated measurements of aneurysm diameter over time (via ultrasound scans), together with information about rupture events (Thompson et al., 2009). Our aim was to develop a model first to estimate the association between the risk of rupture and the size and rate of growth of the aneurysm, and second to make predictions about the future growth and probability of rupture.

## 2 Classical approaches to joint modelling

Suppose longitudinal response data and time-to-event data are available from *m* subjects. Denote 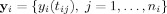 to be a set of longitudinal response measurements for the *i*-th subject collected at times 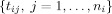. In addition, each subject provides a (possibly right censored) failure time 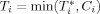 and an event indicator 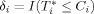, which indicates whether the observed failure time is a true failure time, 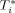, or a censoring time *C*_*i*_.

### 2.1 A survival model with time-dependent covariates

Perhaps, the simplest method to include association between the longitudinal and time-to-event processes is a survival model with the longitudinal measurements specified as time-dependent covariates. The hazard of failure at time *t* for patient *i* is modelled as


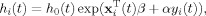


where *h*_0_(*t*) is either a parametric or semi-parametric baseline hazard function, α is a parameter measuring the association between the *observed* longitudinal measurement and the hazard of failure at time *t*, and **x**_*i*_ is a vector of further explanatory variables with regression parameters **β**. Generally, measurements are only available at the examination times *t*_*ij*_ while the model must make assumptions about the value of *y*_*i*_(*t*) continuously throughout time (or at the failure times for the semi-parametric Cox model). Software implementations usually do this by assuming the time-dependent covariates remain constant between examination times, and hence the model for the hazard is


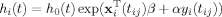


for *t*_*ij*_≤*t*<*t*_*i*,*j*+1_.

The problems associated with this modelling approach are well known as described by Tsiatis et al. (1995). In particular, any true association between the hazard and underlying response will be underestimated due to measurement error (Prentice, 1982). This is problematic if interest lies in understanding the strength of association between the two processes, for example if the response measurement were being considered as a surrogate for failure. Even if decisions are to be based on observed measurements, as is the case with choosing monitoring intervals for AAA, the underlying association between the longitudinal and time-to-event processes is still of importance as it indicates the maximum explanatory power of the longitudinal process that could be obtained by reducing measurement error (Tsiatis et al., 1995). Interpolation of the longitudinal measurements between examination times is a further disadvantage of the time-dependent covariate model, especially if examinations are sparse or taken a considerable time before failure. Such interpolations are a further cause of measurement error, resulting in dilution of the estimated association. Finally, no modelling of the longitudinal process is attempted with this approach, which may also be of interest.

### 2.2 Joint models with shared random effects

To describe the shared random effects model we use notation similar to that presented by Henderson et al. (2000). The response measurements are modelled using a mixed-effects model, a popular and flexible choice for continuous longitudinal data. The model can be written as


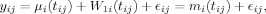


where 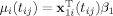 is the mean response for subject *i* at *t*_*ij*_, and is modelled in terms of a set of explanatory variables **x**_1*i*_(*t*) measured at each examination time. Subject-specific random effects are incorporated through *W*_1*i*_(*t*)=**d**_1*i*_(*t*)**U**_*i*_, where **U**_*i*_ is a vector of *q* zero-mean random effects, and **d**_1*i*_(*t*) is a vector of explanatory variables (possibly a subset of **x**_1*i*_(*t*)). The random effects are commonly modelled as multivariate normal random variables, **U**_*i*_ ∼_iid_ *N*_*q*_(**0**,**Σ**). Finally, the ε_*ij*_ ∼_iid_ *N*(0,σ^2^) are the measurement error terms. Thus a linear mixed-effects model with random intercept and slope is obtained by letting μ_*i*_(*t*)=β_11_+β_12_*t* and *W*_1*i*_(*t*)=*U*_1*i*_+*U*_2*i*_*t*.

The hazard of failure is specified by the multiplicative model


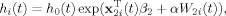
(1)

where **x**_2*i*_(*t*) are a set of explanatory variables with associated fixed effect parameters **β**_2_. *W*_2*i*_(*t*)=**d**_2*i*_(*t*)**U**_*i*_ is a function of the random effects **U**_*i*_ with association parameters **α**. The longitudinal and time-to-event processes can therefore be made stochastically dependent through the use of shared random effects in *W*_1*i*_ and *W*_2*i*_.

Henderson et al. (2000) proposed an extension of this joint model for investigating the relationship between the hazard of failure and the subject-specific intercept, slope, and the current underlying measurement in a linear mixed-effects model. Specifically, this is achieved by letting *W*_1*i*_(*t*)=*U*_1*i*_+*U*_2i_*t* and replacing **α***W*_2*i*_(*t*) in the hazard (1) by 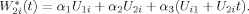 The parameters α_1_, α_2_, and α_3_ measure the association between the hazard of failure and the random intercept, slope and current *W*_1*i*_ value (the subject-level deviation from the mean level at time *t*).

Specialist software is available to fit shared random effects joint models. The JM package in R (Rizopoulos, 2010) allows users to fit a variety of survival models with parametric or semi-parametric baseline hazards, and a wide range of mixed-effects models. The current software can model dependency between the processes using either the current underlying longitudinal value (*W*_2*i*_(*t*)=*m*_*i*_(*t*)), the rate of growth (*W*_2*i*_(*t*)=d*m*_*i*_(*t*)/d*t*) or both. Maximum likelihood estimation (MLE) is performed for parametric survival models, while the EM algorithm is used for Cox-type models. Predicted survival probabilities can be obtained using a Monte–Carlo procedure that samples from an approximation of the posterior distribution of the random effects given an individual's observed data (Rizopoulos, 2010). The software is constantly being updated and readers should refer to http://rwiki.sciviews.org/doku.php?id=packages:cran:jm for new developments. The JoineR collaboration (http://www.liv.ac.uk/joine-r/) also provides R software for fitting a shared random effects model. Here, the association can be linked through either a random intercept *W*_2*i*_(*t*)=*U*_1*i*_, a random intercept and linear slope *W*_2*i*_(*t*)=*U*_1*i*_+*U*_2*i*_*t* or through a quadratic model *W*_2*i*_(*t*)=*U*_1*i*_+*U*_2*i*_*t*+*U*_3*i*_*t*^2^. The software does not currently allow for a parametric specification of *h*_0_(*t*), and standard errors must be obtained through bootstrapping.

### 2.3 A two-stage approach

The final model considered is a two-stage joint modelling approach where the longitudinal process is first fitted separately, and the MLE and best linear unbiased predictors (BLUPs) of the random effects are obtained. Then in a second stage, a model is fit to the survival data using the longitudinal fitted values as covariates. Two-stage approaches have been investigated previously by Bycott and Taylor (1998), Self and Pawitan (1992), Tsiatis et al. (1995), and Dafni and Tsiatis (1998). Let 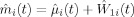 be the fitted value for the *i*-th individual at time *t*, where 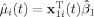 is the fitted mean response and 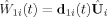 is the fitted individual deviation at time *t*, using the BLUPs of the random effects, 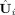.

The second stage of this approach can be conducted as in Section 2.1, using the standard survival analysis software. Since 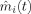 can be evaluated continuously throughout time, the data set can be split into as fine time-intervals as required, so that the assumption of constant longitudinal measurements between examination times is weakened. Note, that for a Cox model, the value of the covariate is only required at the event times, and there is no requirement to split the data set into finer time intervals.

The advantage of using a two-stage approach is that it is relatively quick to implement and requires only standard mixed-effects and survival software. The drawbacks are that it does not correct for event-dependent drop-out, and uncertainty in the estimated MLEs and BLUPs are not carried forward to the survival model, resulting in estimates that are too precise. Furthermore, the form of the BLUPs depends critically on the validity of normally distributed random effects and error terms, an assumption which becomes less satisfactory as time increases and subjects suffer informative drop-out (Tsiatis and Davidian, 2004).

## 3 Classical simulations

To compare the performance of the three approaches described above a simulation study was carried out. These simulations aim to assess to what extent the time-dependent covariate and two-stage approaches adequately model a shared random effects data-generating process and how biased estimates might be (Section 3.1). The joint model is expected to perform well in these simulations. Meanwhile, the robustness of all three approaches under mild and gross model misspecification is assessed in a second set of simulations in Section 3.2.

### 3.1 Performance of time-dependent and two-stage approaches in approximating a joint model

Longitudinal and survival data were generated for 1000 individuals, with follow-up examinations at times *t*=0,2,4,6,8. First, the longitudinal process was simulated from a linear mixed-effects model with the true parameters chosen to be similar to those estimated in the AAA application presented in Section 5. Specifically, the following values were chosen for the parameters: β_11_=38.5 (mean AAA diameter at initial screening in mm), β_12_=1.73 (mean rate of growth in mm/year), σ=2.8, **Σ**_11_=43.5, **Σ**_12_=3.2, and **Σ**_22_=1.7. The time-to-event process was simulated from a time-varying covariate model with constant baseline hazard, and with the hazard at time *t* linked only to the underlying measurement process, *m*_*i*_(*t*). We wished to simulate a relatively rare event, to reflect the small number of ruptures observed in the AAA application. A constant baseline hazard was assumed and fixed at λ=0.008; this corresponds to the hazard of failure when the longitudinal value is equal to the mean of the process over the 10-year follow-up. The association parameter, α, was set to 0.2 to reflect modest correlation between the two processes.

Two censoring mechanisms were considered. First, individuals were subject to random censoring over follow-up, where the time to censoring for subject *i* was simulated from a uniform distribution, *C*_1*i*_ ∼ *U*(0,10). A second censoring mechanism occurred when the subject was first observed to have a measurement over a certain threshold *v*; that is *C*_2*i*_=min(*t*_*j*_: *y*_*ij*_≥*v*, *j*=1,…,6). This mechanism reflects what occurs in the AAA application presented in Section 5, where individuals whose diameter is observed to be above a threshold are referred for surgery. Here, we take *v*=55 mm, to reflect this policy. The observed failure time for each individual was therefore calculated as 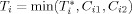, where 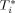 is the simulated true failure time.

All three models were fitted using the same structure as generated the data (i.e. a linear mixed-effects model for the longitudinal process, and a parametric survival model with constant baseline hazard). The time-dependent covariate model used the observed longitudinal values at the examination times as the explanatory variable, while the joint model used the underlying current measurement process and the two-stage model used the fitted values at the examination times (i.e. every 2 years). In addition, we also fitted two-stage models that used fitted values every 6 months, and every month between *t*=0 and the failure/censoring event. Finally, each model was also fitted using a Cox (semi-parametric) baseline hazard. For the two-stage Cox model the fitted values were calculated at the failure times of the simulated data.

We first consider the case where only random censoring is acting on the subjects. Over 1000 simulations, the mean number of failures was 81, with an average follow-up of 4810 person-years (1.7 per 100 person-years). Table 1 shows the mean of the MLEs, average asymptotic standard error, and 95% coverage for the log baseline hazard, log(λ), and the association parameter, α, for the three approaches. Using 1000 simulations the precision (i.e. 95% interval) of the estimated 95% coverages is ±1.5%, while the standard error of the estimates for log(λ) and α was never greater than 0.007 and 0.0005, respectively across all the fitted models. With one exception, discussed below, the mean asymptotic standard errors are in close agreement with the Monte–Carlo empirical standard deviations. The time-dependent covariate model, fitted using streg in Stata, overestimates the baseline hazard by 11% while underestimating the association parameter by 9% (a result of regression dilution caused by using an explanatory variable measured with error). The joint model, fitted using JM in R, provides unbiased estimates of the baseline hazard and association parameter, and good coverage properties when the parametric model is used. A Cox-model, however, provides too small standard errors and hence confidence intervals that are too tight as reflected by the low coverage probability. The mean asymptotic standard error of 0.008 was far lower than the empirical standard deviation of 0.018. Hsieh et al. (2006) showed that standard errors based on the Fisher information are unreliable for semi-parametric joint models due to the implementation of the EM algorithm within the profile likelihood. The solution is to calculate bootstrap estimates of the standard error, although due to computational costs we have not investigated this approach within this simulation study. The two-stage model, using xtmixed and streg in Stata, overestimates the baseline hazard by 13% when fitted values are used every 2 years, but this bias reduces to 4% as the gap times are reduced to monthly. The association parameter is generally underestimated, no matter whether a parametric or Cox model is used, with the parametric model estimates converging to that of the Cox model as the gap times are reduced.

**Table 1:** Results from a simulation study comparing the mean MLE (Mean), mean asymptotic standard error (SE) and nominal 95% coverage of the estimated baseline hazard and association parameter under random censorings

Model	log(λ)=−4.83			α=0.20		
		
	Mean	SE	Coverage (%)	Mean	SE	Coverage (%)
**Time-dependent covariate**						
Constant hazard	−4.30	0.16	10.4	0.186	0.011	72.6
Cox model	–	–	–	0.189	0.014	84.8
**Joint model**						
Constant hazard	−4.85	0.21	95.0	0.202	0.015	95.4
Cox model	–	–	–	0.199	0.008	62.8
**Two-stage model**						
Constant hazard (every 2-years)	−4.21	0.15	4.9	0.195	0.012	90.4
Constant hazard (every 6-months)	−4.56	0.19	61.0	0.194	0.012	89.2
Constant hazard (every month)	−4.65	0.19	76.7	0.191	0.012	85.0
Cox model (at failure times)	–	–	–	0.188	0.015	81.1

The performance of the time-dependent and two-stage models are improved somewhat when both random and threshold censoring are acting on the population (Table 2). In this scenario, the mean number of failures was only 23 over an average follow-up of 4393 person-years (0.5 per 100 person-years). The sparseness of events, resulting in less event-dependent drop-out, could be one reason why the approximate time-dependent covariate and two-stage methods perform better under this scenario. Nevertheless, bias is still present using these approximate methods. The joint model provides unbiased results even under this informative (threshold) censoring scheme. This is because the censoring mechanism causes data to be missing at random (MAR) since drop-out is either random or depends on previously observed data (Little, 1995). This can be adequately dealt with using a likelihood-based joint model.

**Table 2:** Results from a simulation study comparing the mean MLE (Mean), mean asymptotic standard error (SE) and nominal 95% coverage of the estimated baseline hazard and association parameter under random and threshold censorings

Model	log(λ)=−4.83			α=0.22		
		
	Mean	SE	Coverage (%)	Mean	SE	Coverage (%)
**Time-dependent covariate**						
Constant hazard	−4.38	0.20	41.1	0.202	0.041	93.8
Cox model	–	–	–	0.199	0.043	93.2
**Joint model**						
Constant hazard	−4.90	0.25	94.8	0.205	0.040	94.7
Cox model				0.204	0.034	87.4
**Two-stage model**						
Constant hazard (every 2-years)	−4.32	0.20	33.2	0.198	0.036	93.3
Constant hazard (every 6-months)	−4.65	0.21	81.3	0.183	0.032	88.9
Constant hazard (every month)	−4.74	0.18	89.4	0.179	0.031	86.3
Cox model (at failure times)	–	–	–	0.172	0.033	83.2

### 3.2 Performance under model misspecification

**Figure 1:**
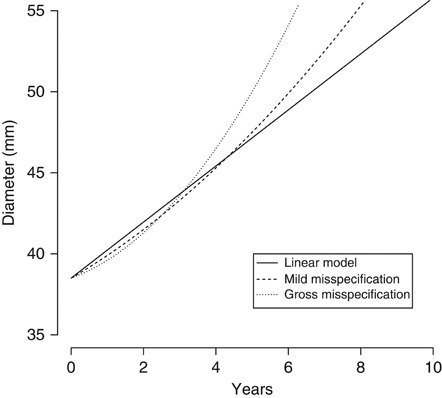
Mean longitudinal profiles of data generated under mild (slight curvature) and gross (large curvature) misspecification. These curves are plotted next to the original linear relationship.

The performance of the three approaches under model misspecification was examined by generating longitudinal profiles that were quadratic in nature, while the models fitted assumed linear trajectories. Figure 1 shows the mean longitudinal profile of data generated under mild (slight curvature) and gross (large curvature) misspecification. These trajectories are plotted next to the linear relationship used in Section 3.1. Under mild misspecification, the following values were chosen for the longitudinal parameters: β_11_=38.5, β_12_=1.3, β_13_=0.1, σ=2.8, **Σ**_11_=43.5, **Σ**_12_=3.2, **Σ**_13_=−0.3, **Σ**_22_=1.7, **Σ**_23_=−0.2, **Σ**_33_=0.08, where β_13_ is the fixed effect for the quadratic term in the model, and **Σ** is a 3×3 between subject variance–covariance matrix for the intercept, linear, and quadratic terms. Under gross misspecification the fixed effect parameters were changed to β_11_=38.5, β_12_=0.8, β_13_=0.3 to reflect greater curvature. All other parameters remained unchanged from the simulations conducted in Section 3.1, while both random and threshold censoring acted on the population.

The results from this simulation study are presented in Table 3. Under both mild and gross misspecification, the time-dependent model severely overestimates both the baseline hazard and the association parameter. The two-stage model overestimates the baseline hazard but underestimates the association parameter. The joint model performs reasonably well, with the association parameter in particular being quite robust to such model misspecification. The baseline hazard parameter is slightly overestimated, and coverage falls to 87% under gross misspecification. Nevertheless, the case for using the joint model is strengthened from these results.

**Table 3:** Results from a simulation study comparing the mean MLE (Mean), mean asymptotic standard error (SE) and nominal 95% coverage of the estimated baseline hazard and association parameter under random and threshold censorings with model misspecification

Model	log(λ)=−4.83			α=0.22		
		
	Mean	SE	Coverage (%)	Mean	SE	Coverage (%)
*Mild misspecification, Mean no. events/f-up=47/4359*						
**Time-dependent covariate**						
Constant hazard	−4.03	0.17	1.8	0.251	0.033	67.7
**Joint model**						
Constant hazard	−4.75	0.22	91.2	0.194	0.027	95.3
**Two-stage model**						
Constant hazard (every month)	−4.51	0.16	48.5	0.174	0.021	73.1
*Gross misspecification, Mean no. events/f-up=101/4157*						
**Time-dependent covariate**						
Constant hazard	−4.01	0.16	1.0	0.251	0.021	29.6
**Joint model**						
Constant hazard	−4.68	0.21	87.2	0.197	0.019	95.1
**Two-stage model**						
Constant hazard (every month)	−4.34	0.14	8.9	0.175	0.015	57.1

## 4 A Bayesian approach to joint modelling

An alternative approach to joint modelling is to use Bayesian MCMC methods. Such an approach has been considered previously by Faucett and Thomas (1996), Brown et al. (2005), Brown and Ibrahim (2003), and Chi and Ibrahim (2006) where tailor-made Gibbs sampling algorithms have been developed. In contrast, Guo and Carlin (2004) use generic MCMC software such as WinBUGS (Spiegelhalter et al., 2003) to implement a joint model. The attraction of using WinBUGS is that a joint model can be coded relatively simply, which applied researchers can then easily adapt to suit their needs. There is also considerable flexibility in model specification. For example, an individual's underlying intercept, slope or current value can all be associated with the time-to-event process. Second, standard errors of the model parameters can be obtained from posterior distributions without the need for asymptotic theory. Finally, in and out-of-sample predictions of both longitudinal and time-to-event processes can be obtained relatively easily using posterior predictive distributions.

However, a current limitation of implementing a joint model in WinBUGS is that, for the time-to-event process, a closed-form evaluation of the integrated hazard function is required in order to formulate the likelihood. For this reason, our Bayesian model is currently restricted to joint models for which the longitudinal process is specified by a linear mixed-effects model, so that *m*_*i*_(*t*)=β_11_+β_12_*t*+*U*_1*i*_+*U*_2*i*_*t* while the hazard for the time-to-event process is modelled using a constant baseline hazard. The baseline hazard is multiplied by a function of the individual-specific intercept, slope and underlying measurement, as follows:


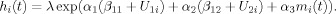


Under this specification the integrated hazard has the following closed-form expression:


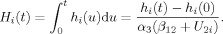


More generally, to relax the assumption of a constant baseline hazard, a piecewise-constant baseline hazard can be specified and the integrated hazard evaluated within each time band separately. Furthermore, to allow for a wider range of hazards and non-linear longitudinal profiles, the trapezium rule could be used to approximate the integrated hazard function, an approach taken by Brown et al. (2005). The WinBUGS code used to implement a constant baseline hazard joint model with linear longitudinal process is given as Supporting Information on the journal website. We use minimally informative Normal(0, 10^4^) priors for all fixed effect parameters (β_11_, β_12_, log(λ), α_1_, α_2_ and α_3_), a Uniform(0, 100) prior for the within patient standard deviation, and a scaled inverse-Wishart prior for the between patient variance–covariance matrix, with degrees of freedom equal to one plus the number of random effects, as suggested previously for the specification of mixed-effect models (Gelman and Hill, 2007).

Inferences concerning future longitudinal values from a new individual (an out-of-sample prediction) can be obtained from the posterior predictive distribution. The existing data on *m* patients can be summarised by 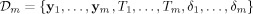 recalling that **y**_*i*_=*y*_*i*_(*t*_*ij*_), *j*=1,…,*n*_*i*_, 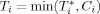, and 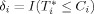. Suppose now that a set of longitudinal measurements are obtained from a new individual, labelled *m*+1, together with information that they have survived up to time *s*. This can be summarised as 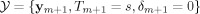. Then, given this data, the predictive distribution for a new observation 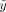 from this individual with random effects 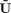 and hyperparameters 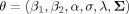 is


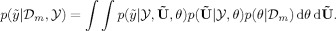


Similarly, the predicted survival probability, for the time to failure 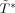 for the new individual, at time *t* given survival up to time *s* is


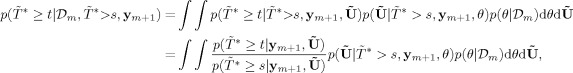


where 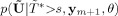 is the posterior distribution of the random effects for the new individual conditional on their data and the hyperparameters **θ**. These quantities are easily obtained within the WinBUGS software, and as demonstrated in the code given as Supporting Information.

## 5 Analysis of the Multicentre Aneurysm Screening Study data

The Multicentre Aneurysm Screening Study (Thompson, 2009) has recorded AAA diameters, using sequential ultrasound measurements, from men aged 65–74 years for up to 11 years. Individuals were considered for elective surgery when their AAA was observed to reach 55 mm or greater in diameter, if their observed growth rate was greater than 10 mm/year or if they were exhibiting symptoms attributable to the aneurysm.

For this analysis the focus is on the growth and rupture rates for the ‘small’ diameter range 30–54 mm. In total, 1122 men whose initial screening diameter was in the range 30–54 mm were included. Rupture events are defined as either an emergency operation for a ruptured AAA, or death from ruptured AAA. Individuals are censored as soon as they are observed to have a measurement ≥55 mm. Censoring also occurs before this time if the individual dies from a non-rupture event, is referred for surgery (for reasons other than their aneurysm being ≥55 mm), is lost to follow-up (LTFU) or is administratively censored. Follow-up for all individuals was restricted to 2 years after their last AAA measurement, since it was not always possible to ascertain whether censoring events (such as operations) were still being recorded for all individuals after LTFU. The administrative censoring date of the database is 31 March 2008. A total of 5281 person-years of follow-up are available for analysis; 20 ruptures occurred, giving a naïve rupture rate of 0.38 per 100 person-years.

**Figure 2:**
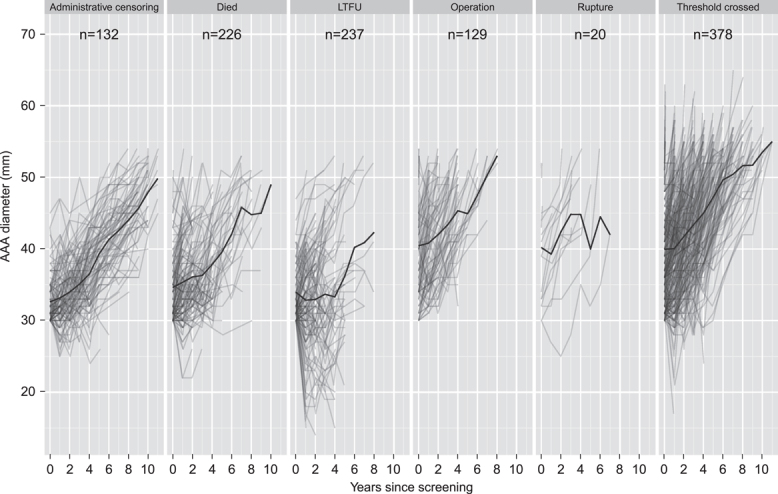
Individual trajectories of AAA growth, stratified by censoring mechanism. The yearly mean AAA diameter is superimposed on the plots.

Figure 2 presents the observed trajectories of the 1122 individuals, stratified by censoring mechanism. The mean AAA diameter is superimposed on each plot; its use is not intended for inferential purposes but to aid discussion of possible informative drop-out. As mentioned in Section 3.1 a threshold censoring mechanism is not problematic since it causes data to be MAR, which can be accommodated using a likelihood-based joint model (i.e. drop-out depends on the *observed* AAA diameter being ≥55 mm) (Little, 1995). Similarly, decisions governing whether the patient should be sent to surgery below 55 mm may have been based on the observed AAA growth rate (92 out of the 129 patients had observed growth of ≥10 mm/year). For individuals who are LTFU, the empirical growth rate is lower. Indeed many individuals are observed to have diameters <30 mm, and essentially the AAA is no longer confirmed in this size range. This may be one explanation why patients drop out of the study. If the reason for LTFU is entirely determined by one or more low measurements then, as before, this is sufficient for an MAR process. Therefore, based on these observations, we assume the data are MAR and further modelling of the missing data mechanism is unnecessary.

A further issue is the competing risk of non-rupture related mortality. Some work has been done to extend the joint modelling framework to the situation of competing risks (Williamson et al., 2008). However, it is not the intention of this paper to address such issues. Rather, we acknowledge that estimates of rupture risk obtained from these analyses are from a non-competing risks model, and reflect risk in the absence of death from other causes.

### 5.1 Models

We begin by fitting joint models where individual growth trajectories are linear. Results from the Classical time-dependent (C-TD), Classical two-stage (C-2S), Classical shared random effects (C-SRE), and Bayesian shared random effects (B-SRE) models, each using a constant baseline hazard, are shown in Table 4, where either an individual's rate of growth or current diameter were used as the explanatory variable in the survival sub-model. In the time-dependent model the observed measurements were used, but the observed rate of growth was found to be too variable to use, with some individuals only having two measurements taken close together. Hazard ratio estimates were generally smaller in the time-dependent and two-stage models. For the C-2S and SRE models, both variables were significantly associated with an increased risk of rupture in univariate analyses. The Bayesian estimate of the hazard ratio for a 1 mm increase in the current underlying AAA diameter was 1.34, 95% credible interval (1.22, 1.49), which is almost identical to a Classical joint model estimate fit using JM. After adjustment in a multivariable model, only the current underlying AAA diameter was found to still have an independent association with rupture, with its hazard ratio remaining relatively unchanged from univariate analyses.

**Table 4:** Hazard ratios (95% confidence/credible interval) associated with the risk of rupture using Classical time-dependent (C-TD), Classical two-stage (C-2S), Classical shared random effects (C-SRE), Bayesian shared random effects (B-SRE) and Classical quadratic shared random effects (CQ-SRE) models, each using a constant baseline hazard

Model	Rate of growth (per mm/y)	Current underlying diameter (per mm increase)	
	*Univariate*		
C-TD	–		1.26 (1.15, 1.38)
C-2S	2.02 (1.56, 2.62)		1.29 (1.16, 1.43)
C-SRE	2.15 (1.54, 2.99)		1.33 (1.21, 1.46)
B-SRE	2.14 (1.49, 3.03)		1.34 (1.22, 1.49)
CQ-SRE	1.83 (1.44, 2.32)		1.31 (1.20, 1.44)
	*Multivariate*		
C-TD	–		–
C-2S	1.29 (0.90, 1.85)		1.27 (1.13, 1.42)
C-SRE	1.39 (0.91, 2.12)		1.30 (1.18, 1.44)
B-SRE	1.36 (0.80, 2.07)		1.31 (1.19, 1.46)
CQ-SRE	1.28 (0.98, 1.69)		1.29 (1.17, 1.42)

The Classical shared random effects model was also extended to allow non-linear individual trajectories through introduction of random quadratic terms. There was strong evidence for non-linear trajectories (*p*<0.0001; likelihood ratio test), although the degree of curvature made little difference to short-term predictions (up to 5 years) of the longitudinal process (Sweeting and Thompson, 2011). The estimated hazard ratios associated with rupture using this Classical quadratic shared random effects (CQ-SRE) model (implemented in JM) are shown in Table 4. The hazard ratios have reduced slightly compared with the linear model, but the effect of current diameter on rupture remains statistically significant. The model was further extended to allow the baseline hazard to be piecewise constant, by using one cut-point placed 5 years after an individual's first screen. There was, however, no evidence of an improved model fit as assessed using the deviance information criteria (DIC).

**Figure 3:**
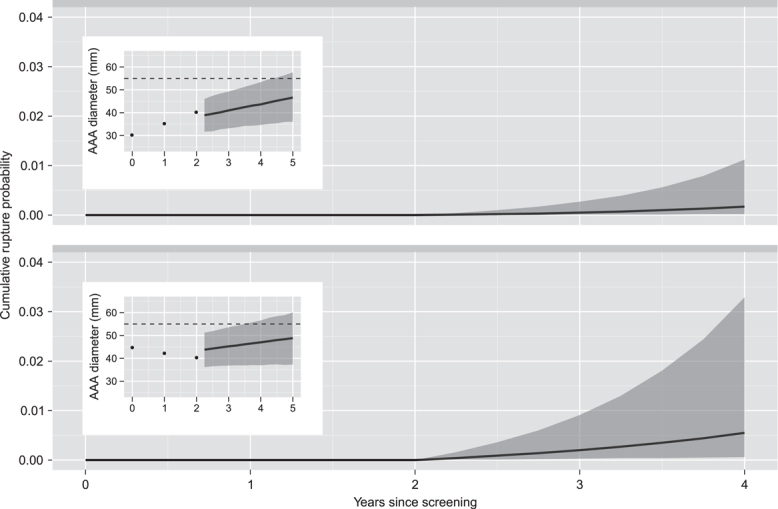
Predicted AAA growth and cumulative probability of rupture for two hypothetical individuals, each with three AAA measurements at years 0, 1, and 2. Cumulative rupture probabilities are shown together with pointwise 95% credible intervals.

Figure 3 shows the predictions of future AAA growth and incidence of rupture for the Bayesian shared random effects model with current underlying diameter as the covariate, a constant baseline hazard, and linear individual trajectories. Two hypothetical individuals are presented; one with measurements of 30, 35, and 40 mm and the other with measurements of 44, 42, and 40 mm, taken at 0, 1, and 2 years after screening. The individuals exhibit observed growth rates of 5 and −2 mm/year with model predicted growth rates of 2.3 and 1.3 mm/year, respectively. Predicted AAA diameter is higher in the second individual due to larger observed diameters, and this is reflected in a higher predicted cumulative risk of rupture. Since small aneurysm ruptures are very rare events, the 95% credible intervals from the model are wide. Nevertheless, the model indicates that to be confident of limiting the probability of rupture to be less than 1%, patient 1 should be re-screened within 2 years (i.e. before year 4) and patient 2 should be re-screened within a year (i.e. before year 3).

## 6 Discussion

Joint models are appropriate when interest lies in the association between a time-dependent covariate measured with error in a survival analysis or when accounting for event-dependent drop-out in a longitudinal analysis. A two-stage approach can provide quick and approximate inferences using standard statistical software. Nevertheless, our simulations have shown that such an approach can often suffer from bias and have poor coverage properties. Furthermore, using the observed longitudinal data as a time-dependent covariate in a survival analysis is not recommended and can result in severe underestimation of the true association, due to regression dilution. Hence, despite its computational burden, a shared random effects model is the recommended approach. Both classical and Bayesian implementations have been developed, the latter being especially useful when survival and longitudinal predictions are required.

AAA screening studies have used various criteria for deciding when to refer patients for elective surgery, based on symptoms, current diameter, and observed growth rate of ≥10 mm/year (Thompson et al., 2009; UK Small Aneurysm Trial Participants, 1995; Scott et al., 1995). In our joint analysis of the Multicentre Aneurysm Screening Study data, despite the small number of ruptures, we have still found a strong association between risk of rupture and underlying current diameter but have found no independent association with an individual's rate of growth. Nevertheless, it should be noted that the current aneurysm diameter and rate of growth are correlated, and detecting independent effects may be difficult for small aneurysms where rupture events are rare. A similar issue is currently the subject of debate in prostate cancer screening, where a high correlation exists between PSA and PSA velocity (rate of change) (Vickers et al., 2011). Using posterior predictive distributions we are able to make predictions of future incidence of AAA rupture. Nevertheless, the uncertainty surrounding such predictions is large, partly due to the rare nature of AAA rupture in the small size range being studied. Care therefore must be taken if basing inferences on the overall risk of AAA rupture from these analyses.

We have estimated a low rupture rate in the aneurysm size range 30–54 mm from this study. This suggests that the current threshold of 55 mm for referral to surgery is relatively safe provided individuals are screened regularly enough to ensure rapid detection when the aneurysm grows this large. The choice of how often the screen individuals should be informed by predictions of AAA growth, and in particular the probability of exceeding 55 mm within a certain time period. This latter prediction can be obtained from the tail area of the posterior predictive distribution of a future AAA diameter, and such calculations have been presented in other research papers (Brady et al., 2004; Sweeting and Thompson, 2011), albeit not through the implementation of a joint model.

## Supplementary material

Detailed facts of importance to specialist readers are published as ”Supporting Information”. Such documents are peer-reviewed, but not copy-edited or typeset. They are made available as submitted by the authors.
